# “Areas of Worklife scale” (AWS) short version (Spanish): a confirmatory factor analysis based on a secondary school teacher sample

**DOI:** 10.1186/s12995-018-0202-0

**Published:** 2018-06-26

**Authors:** B. Masluk, S. Gascón Santos, A. Albesa Cartagena, A. Asensio Martinez, E. Peck, M. P. Leiter

**Affiliations:** 1 Department of Psychology and Sociology, University of Zaragoza, C/ Pedro Cerbuna 12, 50009 Zaragoza, Spain; 2 RedIAPP - Research Network on Preventive Activities and Health Promotion, (RD 12/005/006), Barcelona, Spain; 3 School of Psychology, Deakin University, Post: 221 Burwood Hwy, Burwood, Geelong, VIC 3125 Australia; 4 Psychology Department, Acadia University, 24 Highland Avenue, Box 220, Wolfville, NS B4P 2R6 Canada

**Keywords:** Control, Areas of Worklife, Burnout, values, Confirmatory factor analysis

## Abstract

**Background:**

This study examines the construct validity of the Areas of Worklife Short Scale, a practical instrument to measure employees’ perceptions of their work environments in the sample of secondary obligatory education teachers in Spain.

**Methods:**

Conducted in 33 centers of secondary obligatory education in Spain (*N* = 677). Confirmatory Factor analysis for 3 different models for the 29-items version and 1 model for the 18-items version was tested.

**Results:**

Results confirmed that the short AWS short version had the best fit to the data than any other model proposed (GFI-Satorra-Bentler scaled chi-squared = 320.19, × 2/*df* = 2.337) and good fit indices (CFI = 0.911; RMSEA = 0.046).

**Conclusions:**

This analysis ultimately supports the appropriateness of AWS short version to explore areas of worklife and therefore can indicate the factors that contribute to burnout in the sample of secondary obligatory education teachers in Spain. Therefore it has been confirmed that this tool is able to assess the 6 domains of work environment of secondary schools teachers.

## Background

### Burnout

Retaining well-qualified teaching professionals is a growing concern as burnout and work-related illnesses are reducing the number of highly capable workers.

According to Maslach and Jackson [1] the exposure to chronic stress may lead to burnout, a “psychological syndrome of emotional exhaustion, depersonalization, and reduced personal accomplishment, which can occur among individuals who work with other people in some capacity”. E.g. Gascon et al. used that definition [2] (p.2): “The authors understand burnout to be the most serious consequence of job stress, when all coping strategies have failed and the individual feels emotionally drained, unconnected to their work and useless.” Other authors use other definition of stress like Demerouti et al. [3] (p 501) which use the term “stressor” only when an external factor has the potential to exert a negative influence on most people in most situations.

These authors refer to the definition of stress of Lazarus & Folkman and McGrath [4, 5] according to which stress is defined in terms of a disruption of the equilibrium of the cognitive-emotional-environmental system by external. Thus, external factors known as stressors could also lead to a state of well-being, as long as the person in question has adequate coping and performance capabilities. On the other hand, international standards, including those of the European Union, have been based on the Plath and Richter [6] model, which establishes that stressors are one of the short – term consequences of strain at work (among others such satiation, monotony and mental fatigue).

Following this conceptualization, stressors are understood as complex psychosomatic reactions to situations of overload or underload, causing frustration of personal goals and feelings of discomfort and tension [6, 7]. A prolonged exposure to stress situations would lead to the continuous feeling of stress, exhaustion and, finally, health problems.

Teachers are frequently confronted with interpersonal processes, as they work in environments of constant interaction with fellow teachers, students, and parents. Additionally, they are the source of interpersonal conflict management between their students on a daily basis. Teachers therefore constitute a specific sample of employees, among mangers and nurses, who experience higher levels of work-related stress, in comparison with other groups [8, 9]. Teachers are continuously exposed at external stressors [10], interpersonal factors in particular, which influence negatively their health [11, 12].

Kyriacou [13] (p. 28) defines the teacher’s stress as an “*experience by a teacher of unpleasant, negative emotions, such as anger, anxiety, tension, frustration or depression, resulting from some aspect of their work*”. He additionally found that teachers had low levels of well-being caused by many workplace factors. These workplace factors included time spent at work, level of workload, ability to manage change, student behavioural problems, student motivation, being evaluated by others, role conflict and ambiguity, poor working conditions, self-esteem and status [13]. The factors leading to low levels of well-being among teachers have been identified and the relationship between teacher’s stress and job satisfaction [14] is well known. Therefore it is an important next step in the research to better understand the factors that are behind teacher’s wellbeing.

### Demand – Control model

In the demand-control model of stress [15], job demands constitute the main stressor, which increases when the individual has low levels of perceived control over their work situation. Job demands, refer to an employee’s workload, which have been defined as the amount of work that needs to be done, time pressures and conflicting demands. The idea of control derived from Demand - Control model [16] studies the importance of being able to make decisions at work. This notion is supported by other empirical studies, which have found that job autonomy is crucial for the health of employees. This model also served as the basis for Leiter and Maslach [17] (p. 59) for the ideation of the questionnaire of six areas of worklife where the Demand – Control model is reflected in the area of workload and control.

### Six areas of Worklife

Leiter and Maslach [18] identified six areas or - sub-scales of the work environment as most relevant to the relationships people develop with their work. The first area is workload, which represents the number of hours worked, the amount of time needed to recover after work, and the nature of workload one carries (heavy, light, difficult, dangerous etc.). The second area of worklife is control. Control at work encompasses employees’ perceived capacity to influence decisions that affect their work and access to the resources that enable them to develop professionally. The third area of worklife is reward and recognition, which is characterized by adequate pay, appreciation from service recipients or supervisors, promotion prospects, and other forms of recognition. The fourth area of worklife is community, which assesses integration within the team, mutual trust, and the overall social network within the workplace. The fifth area of worklife is fairness, which represents discrimination, favouritism, and other employee perceptions of fairness in the workplace. The last area of worklife is values, which measures the extent to which one’s personal values align with their organization’s values.

The Areas of Worklife Scale (AWS) has been designed with the objective to assess the workplace within the context of organizational interventions both for researchers and practitioners. In this model, the level of perceived balance between the person and the job is the key point in developing better adaptation [18].

The scale has been recently translated into Spanish and validated [2] in a sample of health professionals and therefore can be used in Spanish-speaking countries to assess job stressors that contribute to burnout. However, there is no validation with the sample of teachers in Spain to date so that being the first study to verify the factorial structure of this questionnaire in education professionals allows us to investigate the usefulness of this test to measure burnout teacher.

The survey is comprised of 29 items. It can also be used together with the Maslach Burnout Inventory General Survey (MBI-GS); [19] a questionnaire of 16 items that provide information on the three dimensions of the burnout-engagement continuum: exhaustion-energy; cynicism-involvement; and inefficacy-efficacy. Gascón et al. [2] has used MBI-GS to evaluate its concurrent validity of Spanish version of AWS.

### Workload and control

The areas of workload and control are based on Demand-Control model of job stress [15, 20]. Many studies have shown [21], recently Nishimura et al. [22], that increased workload has a strong relationship with the exhaustion dimension of burnout. In fact, Leiter and Maslach [18] (p.96) note that “A sustainable workload stops the cycle of exhaustion…(and) is a driving force in the experience of burnout for many people”.

The changes in recent years have made a modification in the perception of teachers, who apart from their traditional functions also have an important role to promote healthy behaviors and actively collaborate in the tasks of attention to students with specific educational needs, among others. Some authors use the term intensification to refer to this extension of the task range that now encompasses a multitude of actions [23].

Regarding control, it is generally accepted in the field of organizational psychology that job stressors tend to reduce the individual’s capacity to exert the control over ones work. Likewise, the conservation of resources theory of stress [24] maintain that burnout is more likely to occur when certain resources, control amongst others, are lost or inadequate to meet the demands. Studies have found that stress-related outcomes can be improved by increasing people’s control over their work [25], once again pointing to the strong relationship between sense of control and stress. Ballet and Kelchtermans [23] explain that the experience of intensification is largely characterised by a loss of control.

#### Community

A sense of community is derived from a positive social environment with no office politics and incivility. Community has been primarily described in terms of social support received from supervisors, coworkers, and networks of family and friends [26]. It is associated with greater engagement [27] and exhaustion [28]. As demonstrated in Bakker [29] social support is one of the most important predictors of extra-role performance, through its relationship with the disengagement component of burnout.

#### Reward

Reward represents a meaningful reward system in place for employees. Specifically, the reward can be verbal recognition or even monetary rewards, such as bonuses. It also gives clear indications of what the organization values are [30]. Employees will experience the most balance when they are rewarded adequately for the effort they put into their job. This is consistent with Siegrist effort-reward imbalance model [31]. Studies have also found that insufficient reward is strongly related to burnout [32].

#### Fairness

Employees with high perceptions of fairness have been described by Leiter and Harvie [33, 34] as individuals who find that there is little to no injustice within their workplace, no unfair promotions, and no favouritism. They also purpote that fairness is related to burnout. Specifically, they state that supervisors who are both fair and supportive induce more acceptance of major organizational change and their subordinates are less susceptible to burnout.

Employees who perceive their supervisors being both fair and supportive are less susceptible to burnout, and are more accepting of major organizational change [33]. Fairness can be explained by the effort-reward-imbalance model [35]; a perceived imbalance between high efforts spent and low rewards received leads to high impact of adverse health effects.

#### Values

Strong values alignment indicates that the same things that give employees a sense of accomplishment at work are also valued by their organization. The professions in which the commitment to work is essential, should reflect them in the organizational mission which will be followed by the organization and the worker. This incompatibility of values between the organization and the employee has been found to increase the occupational burnout and decrease the work engagement [36]. Conflict in values is related to all dimensions of burnout [33]. Also has been found that the value congruence of employees with the organization has more impact on job satisfaction than the value congruence among co-workers [37].

### Research purpose

The aim of the study was first to confirm the structure of the original six AWS factors in a sample of compulsory secondary school teachers in Spain. The second objective was to validate the short version AWS scale on a sample of secondary compulsory teachers.

## Method

### Sample

The sample has exceeded the usual rule of 10 participants per survey item, and was comprised of 677 secondary school teachers selected from 33 centres, who met the following criteria: teachers of the secondary obligatory education and teachers who had remained in their current job for at least a year. Participants whose questionnaires were incomplete (*N* = 63) were excluded from analyses. The average age of participants was 45.07 years (*SD* = 9.8), 58% had 12 years of tenure or more, and there were approximately equal numbers of men and women (and 51.9% women, 48.1% men). The sex distribution data are representative taking into account official statistical data provided by the Ministry among secondary school teachers: 40.3% were men and 59.7% were women [38]. In terms of age, and based on the same data source, the largest group is of teachers whose ages are between 40 and 49 years old with 37.1% and followed by the group with ages between 50 and 59 years (35%) which suggests that the sample is representative. No official data has been found in terms of tenure of teachers in Spain, and therefore the results obtained in this variable should be generalized with caution.

Most were married or living with a partner (94.2%) and 71.7% had children. Most were working in urban environments (81%) and in public settings (89.7%). Based on the data provided by the Ministry of Education and Culture of the Government of Spain, in the year 2014–2015 there have been 171.683 teachers of primary and secondary education and professional training [38].

### Measures

The survey included the AWS questionnaire, consisting of 29 questions, a sociodempographic questionnaire, and an occupational factors questionnaire. The questionnaires collected information on gender, age, marital status (or stable relationship in general), children (the number of), dependants (number of), tenure in education and in the actual workplace, contract type (public official vs. resident vs. temporary worker), profession, type of school (public vs. private), and environment (urban vs. rural).

#### Manageable workload

Three questions were used to assess participants’ levels of manageable workload. Participants were asked to rate how much they agreed with the statements on a 5 point scale (1 = Strongly Disagree, 2 = Disagree, 3 = Neither Agree nor Disagree, 4 = Agree, 5 = Strongly Agree). An example item: “I do not have time to do the work that must be done.”

#### Control

Three questions were used to assess participants’ levels of control at work. Participants were asked to rate how much they agreed with the statements on the 5 point scale. An example item: “I have control over how I do my work.”

#### Reward

Three questions were used to assess participants’ levels of reward at work. Participants were asked to rate how much they agreed with the statements on the 5 point scale. An example item “I receive recognition from others for my work”.

#### Community

Three questions were used to assess participants’ levels of reward at work. Participants were asked to rate how much they agreed with the statements on the 5 point scale. An example item: “People trust one another to fulfill their roles.”

#### Fairness

Three questions were used to assess participants’ levels of reward at work. Participants were asked to rate how much they agreed with the statements on the 5 point scale. An example item: “Resources are allocated fairly here”.

#### Values

Three questions were used to assess participants’ levels of reward at work. Participants were asked to rate how much they agreed with the statements on the 5 point scale. An example item: “My values and the organization’s values are alike.”

The North American norms of both the MBI and the AWS have been established by Leiter and the Spanish norms have been established by Leiter et al. [39]. Table 1 shows the Spanish norms obtained in the study with Spanish nurses.

**Table 1:** “Main characteristic description from AWL in the Spanish version”, comparison of Spanish and Canadian norms

Measure	Mean	S.D.	t	p-value
Workload	3.06	0.83	9.36	< 0.001
Control	2.73	0.91	−4.45	< 0.001
Reward	3.00	0.82	−6.68	< 0.001
Community	3.19	0.82	−8.07	< 0.001
Fairness	2.54	0.72	−8.93	< 0.001
Values	3.01	0.70	−14.52	< 0.001

*N* = 834 for Spanish sample

### Statistical analysis

SPSS 20.0 was used for all analyses. Results considered statistically significant if their *p*-values were < 0.05. To study the construct validity, the results of a factor analysis using principal components analysis with VARIMAX rotation were explored.

The analysis was started by conducting a scree test to obtain principal components extraction. Next the eigenvalues were examined with the criteria of 1.0 or greater [40] Table 2 shows the eigenvalues and percentage of explained variance associated with each factor.These criteria indicated the suitability of 6 factors for rotation. The final results indicated that the 6-factor solution produced the highest factor comparability coefficients.

**Table 2:** “Eigenvalues and percentage of explained variance associated with each factor”

	Eigenvalue	Percentage explained variance
Factor 1	6.66	22.98%
Factor 2	2.55	8.81%
Factor 3	1.82	6.28%
Factor 4	1.55	5.34%
Factor 5	1.41	4.86%
Factor 6	1.25	4.34%

Based on these results, an exploratory factor analysis (EFA) and Varimax rotation for 6 factors of 29-item AWS was tested. Table 2 shows the factor loadings obtained in the Principal Components Factor Analysis (PCFA). Table 3 presents factor loadings on each item.

**Table 3:** “Principal components factor analysis (PCFA) of Spanish version”

	Workload	Control	Rewards	Community	Fairness	Values
(Item 1) Workload 1 - Classroom workload	**0.65**	− 0.37	− 0.09	− 0.19	0.10	0.30
(Item 2) Workload 2 - Intensity and workload	**0.61**	− 0.07	0.00	−0.09	0.12	0.26
(Item 3) Workload 3 - Fatigue	**0.77**	−0.05	−0.31	− 0.11	0.16	0.38
(Item 4) Workload 4 -Interference with personal interests	**0.70**	−0.19	−0.14	− 0.13	0.07	0.26
(Item 5) Workload 5 -Time pressure	**0.53**	−0.39	−0.06	− 0.06	0.53	0.20
(Item 6) Workload 6 -Freetime disconnection	**0.44**	−0.38	−0.15	− 0.14	0.44	0.10
(Item 7) Control 1 -Control over one’s tasks	0.30	**−0.70**	−0.14	− 0.22	0.07	0.11
(Item 8) Control 2 -Influence on the relevant aspects of work	0.25	**−0.73**	−0.31	− 0.15	0.22	0.27
(Item 9) Control 3 -Professional autonomy	0.26	**−0.62**	−0.34	− 0.37	0.13	0.17
(Item 10) Rewards 1 -Positive feedback	0.34	−0.26	**−0.73**	− 0.29	0.34	0.3
(Item 11) Rewards 2 -Appreciation	0.14	−0.19	**−0.69**	− 0.32	0.14	0.33
(Item 12) Rewards 3 - Lack of recognition	0.14	−0.21	**−0.81**	− 0.19	0.14	0.17
(Item 13) Rewards 4 - General recognition of one’s efforts	0.26	−0.21	**−0.81**	− 0.22	0.26	0.20
(Item 14) Community 1 - Trust within a group	0.32	−0.25	−0.14	**− 0.74**	0.32	0.23
(Item 15) Community 2 - Support within a group	0.31	−0.43	−0.12	**− 0.09**	0.27	0.12
(Item 16) Community 3 - Cooperation within a group	0.01	−0.23	−0.20	**− 0.79**	0.45	0.36
(Item 17) Community 4 - Communication	0.15	−0.15	−0.25	**− 0.82**	0.37	0.27
(Item 18) Community 5 - Group closeness	0.23	−0.08	−0.42	**− 0.62**	0.15	0.11
(Item 19) Fairness 1 - Resource allocation	0.5	−0.17	−0.07	− 0.31	**0.69**	0.30
(Item 20) Fairness 2 -Merit – based system	0.06	−0.11	−0.07	− 0.21	**0.34**	0.26
(Item 21) Fairness 3 - Fairness in appeal procedures	0.12	−0.33	−0.16	− 0.43	**0.60**	0.38
(Item 22) Fairness 4 - Justice – based leadership	0.09	−0.20	−0.20	− 0.41	**0.84**	0.26
(Item 23) Fairness 5 - Favoritism in decisions	0.16	−0.15	−0.27	− 0.22	**0.80**	0.20
(Item 24) Fairness 6 - Favoritism and career	0.1	−0.09	−0.27	− 0.19	**0.72**	0.10
(Item 25) Values 1 - Value fit	0.19	−0.11	−0.21	− 0.23	0.20	**0.80**
(Item 26) Values 2 - Organization’s goals influence	0.07	−0.17	−0.15	− 0.14	0.23	**0.72**
(Item 27) Values 3 - Objective’s fit	0.47	−0.16	−0.24	− 0.27	0.20	**0.79**
(Item 28) Values 4 - Quality within the organization	0.05	−0.27	−0.09	− 0.40	**0.60**	0.22
(Item 29) Values 5 - Endargenment of values	0.11	−0.02	−0.03	− 0.39	**0.45**	0.32

## Results

All six subscales of the AWS have been found to be highly reliable, with Cronbach alpha’s ranging from .70 to .82 [18]. In the current study the Cronbach’s Alpha for the long version ranged from 0.66 to 0.80: Workload =0.70; Control =0.66; Reward =0.80; Community = 0.70; Fairness = 0.77; Values = 0.66.

Items that didn’t load on their corresponding factors were values item number 4; “This organization is committed to quality”, and values item 5; “Working here forces me to compromise my values”. These two items loaded instead onto the fairness factor.

### Construct validity

The next step was to analyze the construct validity using confirmatory factor analysis (CFA) by using EQS for Windows (version 6.1) with the robust analysis option. The skewness ranged from .35 to − 1.12 and kurtosis from 1.25 to −.88, both within acceptable ranges.

### Factor replication

The proposed model assigned the 29 AWS items to the six factors in accordance with the standard practice. The six factors were freed to covary. None of the error correlations were freed. The initial item within each subscale was fixed at 1.00 to establish scale. The initial analysis indicated problems with item Workload 6 (“I leave my work behind when I go home at the end of the workday”): it was not compatible with any of the scales. This item was therefore deleted from all analyses, as subsequent analyses were conducted with the remaining 28 items.

### Model evaluation

To determine how the model represented the data the goodness-of-fit indicator (GFI) - Satorra-Bentler scaled chi-squared and × 2/df were used. The covariance fit index (CFI) as well as the root mean square error of approximation (RMSEA) were also applied. The model of fit was evaluated based on the Hu and Bentler’s [41] guidelines of CFI greater than .90 or greater is considered a good fit, and a CFI of .95 or greater is considered an excellent fit.

The initial analysis for **Model 1** indicated a poor fit (Satorra-Bentler scaled chi-squared = 1004.251004.25; × 2/df = 2.99; CFI = 0.838; RMSEA = 0.057). Modification indices indicated problems with two items: values items 4 and 5. These items had modest coefficients (Values4—.440; Values5—.414) and large modification indices indicating that the items fit better with the Fairness area of worklife factor than the Values factor (Values4—134.96; Values 5—40.20).

A **Model 2** that reassigned Values4 and Values5 to Fairness produced a better fit, but a fit that failed to reach an acceptable criterion (Satorra-Bentler scaled chi-squared = 838.75; × 2/*df* = 2.50; CFI = 0.878; RMSEA = 0.050). The reassigned items had improved coefficients (Values4—.590; Values5—.439) and the Modification Indices for assigned these items to the Values factor were small.

The modification indices indicated problems with some correlated errors. As found in Gascon et al. [2] the problems were largely with sequential items within a given subscale.

A **Model 5** with five freed error terms for sequential items (Reward3, Reward4; Fairness2, Fairness3; Fairness3, Fairness4; Fairness4, Fairness5; Values4, Value5) produced an acceptable fit (Satorra-Bentler scaled chi-squared = 739.04; 330 df; × 2/*df* = 2.240; CFI = 0.901; RMSEA = 0.045).

In the light of the results of the CFA of 3 models explained above, the decision was made to evaluate the goodness-of fit and the fit indices in the short version of AWS.

### The AWS short scale

The Areas of Worklife Scale short survey provides the information about the six sub-scales or areas of the worklife just as the original Areas of Worklife Scale. The items for the short version were chosen with 2 considerations: items with the highest factor coefficients and low error correlations among the items within a factor.

It is comprised of 18 items that have been extracted from original AWS. In the workload subscale items 1, 2 and 4 are retained. In the control subscale items 7, 8 and 9 are retained. For the reward subscale items 10, 11 and 12 are retained. In the community subscale items 15, 16 and 17 are retained. For the fairness subscale items 19, 20 and 21 are retained. For the values scale items 25, 26 and 27 are retained.

Some of the six subscales include only positively worded items e.g. (control), “I can influence management to obtain the equipment and space I need for my work.”, and some include both positively and negatively worded items e.g. “My efforts usually go unnoticed” (reward).

The CFA analysis of the short AWS version **(Model 4)** shows an acceptable goodness-of-fit (GFI-Satorra-Bentler scaled chi-squared = 320.19, × 2/*df* = 2.337) and good fit indices (CFI = 0.911; RMSEA = 0.046).

Table 4 shows the Goodness of fit indices (robust analysis option) for the Confirmatory Factor Analysis for all tested models.

**Table 4:** “Goodness of fit indices (robust) for the Confirmatory Factor Analysis”

Model	Satorra-Bentler ×2	df	×2/df	CFI	RMSEA
Model 1: Items 28 & 29 on Values	1004.25	335	2.99	.838	.057
Model 2: Items 28 & 29 reassigned to Fairness	838.75	335	2.50	.878	.050
Model 3: Items 28 & 29 reassigned to Fairness with 5 freed errors terms	739.04	330	2.24	.901	.045
Model 4: Short version with workload 6 item	320.19	137	2.33	.911	.046

**Fig. 1:**
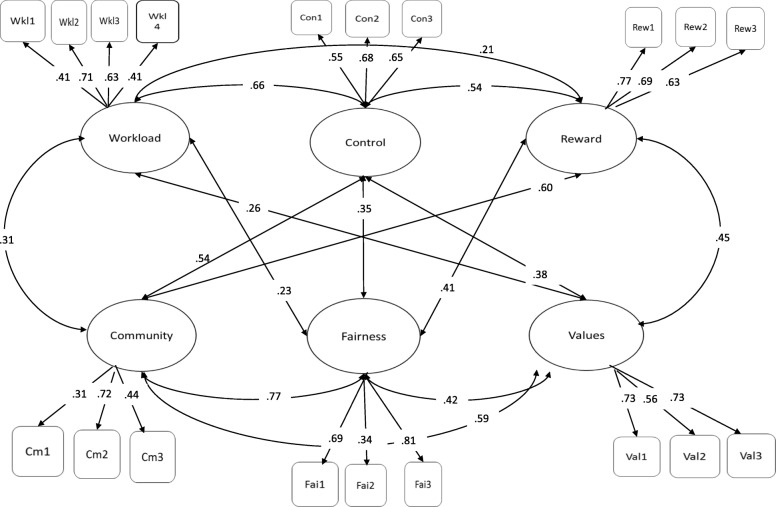
Correlations between factors and assignment of items to the appropriate subscales in the six-factor solution. *N* = 677

The assignment of items to the appropriate subscales in the six-factor solution as well as the correlations between factors is displayed in Fig. 1. All coefficients between factors were significant.

## Discussion

Our results confirmed that the short version of the AWS had the best fit to the data compared to any other model proposed. In the initial analysis there were problems with one item on the workload sub-scale (“I leave my work behind when I go home at the end of the workday”), which was deleted from further analysis of the long AWS version but added again in the short AWS model analysis.

The items of the long version that seemed to make the difference between Spanish health workers and Spanish secondary education teachers were: “This organization is committed to quality”, and “Working here forces me to compromise my values”, both belonging originally to the sub-scale of values and loading instead onto the fairness sub-scale. The question remains whether values and fairness are components of the same dimension, rather than two distinct factors. Nevertheless, numerous studies [2, 18, 42] confirm the six-factor structure of AWS. This suggests that teachers may be most likely to perceive their commitment to quality and value consistency in terms of justice – rather than values. Yet, the interprofessional difference in perceiving the justice and values has rarely been studied in detail.

### Limitations

We are aware that one of the limitations of the current study is that the data are not based on a representative sample. These concerns should be reflected in the interpretation of the results of this survey and broad generalizations about the entire population shouldn’t be made. Also, there were no other work-life questionnaires available to compare our results with. It would be an interesting exploration for future research to analyze the mediation model between the AWS short version and a standard burnout measure, as was conducted by Gascón et al. [12] who made the Structural Equation Model analysis of areas of worklife and burnout factors measured by Maslach Burnout Inventory General Survey [19].

## Conclusions

The structure of the original six AWS factors has been confirmed in a sample of compulsory secondary school teachers in Spain. The second objective, which was to validate the short version AWS scale on a sample of secondary compulsory teachers was been accomplished. Therefore it has been confirmed that this tool is able to assess the 6 domains of work environment of secondary schools teachers. It also allows researchers to identify the areas in which the potential risk of burnout is high, which should be the object of special attention and regular assessment.

This is the first study that provided information about the psychometric properties of the Areas of Worklife Scale short version, and the first scale to assess the relationships of secondary school teachers and their organizations.

In the PCFA 2 values items has been moved on fairness scale and one workload item has been deleted. Our recommendation is to use the 28 – item version (without workload 6 item) but average the problematic values items on fairness scale. The short scale should be used as a first-choice measure when used in educational environment in Spanish-speaking countries.

The applicability of this tool is high due to its reduced size and the specific design to be used in preventive policies by organization boards. It ought to be taken into account that current evaluations of the work environment and the burnout risk at work should be an ongoing task, which requires the rationalization of resources such as time spend on fulfilling the survey. The AWS is a reliable measure for assessing quality of worklife in organizations as well as designing intervention programs. A possible intervention preceded by the assessment with the AWS could focus on enhancing value congruence targeting corporate communication or increasing the control over one’s work.

Finally, it is suggested that further studies should be conducted in order to evaluate the concurrent validity with other questionnaires.

## Abbreviations

AWS: Areas of Worklife Short Scale
CFI: The covariance fit index
EFA: Exploratory factor analysis
EQS: Structural Equation Modeling Software
GFI: Goodness-of-fit indicator
MBI-GS: Maslach Burnout Inventory General Survey
PCFA: Principal components factor analysis
RMSEA: Root mean square error of approximation
